# The currently available diagnostic tools and treatments of scabies and scabies variants: An updated narrative review

**DOI:** 10.1097/MD.0000000000033805

**Published:** 2023-05-26

**Authors:** Jacob Al-Dabbagh, Razan Younis, Nemat Ismail

**Affiliations:** a Faculty of Medicine, Tishreen University, Latakia, Syria; b Faculty of Medicine, Tartous University, Tartous, Syria.

**Keywords:** infestation, parasitism, pruritus, *Sarcoptes scabiei*, scabies

## Abstract

Scabies is a neglected tropical disease that continues to have global impacts and long-term health consequences. It is caused by the mite *Sarcoptes scabei* var. hominis, which is an obligate ectoparasite that lives in the epidermis of the human skin. Scabies is common in poor communities due to overcrowding in places like old age homes, prisons, and homeless and displaced children. However, developed countries are also susceptible to scabies infestations, such as in institutional outbreaks or small epidemics under war conditions or during natural disasters. The diagnosis of scabies may be assisted by invasive and noninvasive tools; However, the history and examination findings are usually adequate to confirm the clinical suspicion. Here, we present an updated review of scabies by focusing on the diagnostic approaches, treatment, and prevention of scabies.

## 1. Introduction

Human scabies is a neglected tropical disease of the skin that affects all age groups.^[1,2]^ It is a highly contagious skin infection that presents as generalized pruritus and is caused by the *Sarcoptes scabiei (S scabiei*) var. hominis mite, which is a constrained ectoparasite that lives in the epidermis.^[3–5]^

Younger populations, particularly children aged 1 to 4 years, are more affected by scabies in tropical and low/middle-income nations.^[6,7]^ On the other hand, the prevalence of scabies is more evenly distributed across all age groups in high-income nations.^[7]^

Globally, scabies affects 200 to 300 million people per annum, with a prevalence of infections occurring in resource-poor tropical regions.^[2,8]^

Multiple family members can be simultaneously affected as scabies mites are transmitted by direct and indirect transmission.^[9]^ Indirect transmission via fomites plays an important role in crusted scabies.^[4,10]^ However, recent studies suggest that indirect transmission is negligible and improbable to play an essential role in classic scabies.^[7]^ Scabies is also known to be a sexually transmitted infection.^[4]^ The long period and frequency of direct skin-to-skin contact, as well as the number of mites on the skin, increase the risk of scabies transmission.^[8]^

This article seeks to summarize the available diagnostic tools and treatment of scabies and provides an updated review of the understanding of scabies variants.

## 2. Clinical presentation

### 2.1. Scabies variants and manifestations

#### 2.1.1. Classic scabies.

Classic scabies is expressed by erythematous papular eruptions, burrows, and pruritus.^[8,11]^ The papules are numerous and generally measure 1 to 2 mm in diameter.^[8]^ Some of the papules may be crusted, scaling, or excoriated.^[8]^ The burrows are presented as whitish, grayish, reddish, or brownish serpiginous linear tracks that are elevated and end with an intact vesicle or erosion involving the mite.^[4,8]^ They measure about 0.5 millimeters wide and a few millimeters in length in the superficial epidermis.^[8]^ Pruritus is the dominant symptom of scabies, which can be severe and may have a negative impact on quality of life.^[6,7]^ Typically, the pruritus is generalized and worse at night and after taking hot showers.^[8,11]^

The nocturnal pruritus may be explained by the mites’ increased activity at night.^[12]^ Their vigorous movement can cause allokinesis and/or hyperkinesis and the feces and/or eggs produced alongside is able to cause itching.^[12]^ However, pruritus may be not present in infants, the elderly, patients who receive topical corticosteroids in an inappropriate manner, or patients who undergo immunosuppressive/anti-inflammatory treatment.^[6]^

Symptoms typically occur 2 to 6 weeks after the initial infestation, but in cases of reinfestation, the symptoms can occur as early as 1 to 3 days.^[4,6,7]^ The lesions are usually distributed symmetrically and are observed on the interdigital spaces, the volar surface of the wrists, soles of the feet, lateral aspects of fingers, lateral and posterior aspects of the feet, extensor of the elbow, areola (in women), buttocks, genitals, axillae, umbilicus, knees, groins, and thighs.^[7,8,13]^ The neck and scalp are typically excluded locations for scabies, except in immunocompromised patients, infants, the elderly, and crusted scabies.^[4,13]^ Anyhow, the scabies mites evade locations that have a high density of pilosebaceous follicles and sebaceous glands.^[4,8]^

Disseminated erythematous papules, linear scratch marks, excoriations, hemorrhagic crusts, eczema, vesicles\bullae, and pustules and impetigo from secondary bacterial infection are seen in severe cases of scabies.^[13]^

#### 2.1.2. Crusted scabies.

Crusted scabies is characterized typically by hyperkeratotic dermatosis.^[7,8]^ It is commonly seen on the soles, palms, ears, and extensor surfaces of the elbows, but may present anywhere on the body.^[4,8]^ On the other hand, restricted crusted scabies can be presented on the scalp, face, fingers, toes, toenails, soles, and genitalia.^[6]^

The surface of the infectious skin appears smooth, velvety, and red when the crusts are removed with pruritus which may be mild or absent.^[4,8]^ It is common to observe generalized lymphadenopathy, peripheral blood eosinophilia, and elevated serum IgE levels in crusted scabies.^[7,8]^ Secondary bacterial infection is also common and associated with remarkable mortality.^[7,8]^

Crusted scabies essentially occurs in individuals with defective T-cell immunity (such as HIV, leukemia, and lymphoma) and who have reduced cutaneous sensation (such as leprosy and neurological disorders) and reduced capability to debride the mites mechanically (such as critical illness, senile dementia, and Down syndrome).^[4]^ However, it is possible to occur in the absence of these risk factors.^[6]^

Davis et al developed a severity grading scale for crusted scabies based on the distribution and extent of the crusts, severity/depth of the crusts, the number of past episodes for crusted scabies, and the degree of skin cracking and pyoderma (Table 1).^[4,7,13]^ The combined scores from each domain, which range from mild to severe, are as follows: grade 1 (score 4–6), grade 2 (score 7–9), and grade 3 (score 10–12).^[7]^

**Table 1:** The grading scale for crusted scabies.

Clinical assessments	Mild	Moderate	Severe
Distribution and extent of the disease	1	2	3
Severity/depth of the crusts	1	2	3
The number of previous episodes (hospitalizations)	1	2	3
The degree of skin cracking	1	2	3
Pyoderma	1	2	3
Grade	1 (4–6)	2 (7–9)	3 (10–12)

#### 2.1.3. Nodular scabies.

Nodular scabies, a clinical variant of scabies, can manifest as skin-colored, reddish-brown, or violaceous nodules that can occur as a result of an exaggerated hypersensitivity reaction or from rubbing and scratching and often persist months after treatment.^[6,13]^ Nodular scabies often affects infants and young children and may localize on the axillae, trunk (in infants), groin, and male genitalia.^[6,14]^

#### 2.1.4. Bullous scabies.

Bullous scabies, a rare clinical variant of scabies, usually affects elderly patients and manifests as extremely itchy bullae that can be tense or flaccid, with or without classic scabies lesions.^[4]^ The trunk and extremities are the most common predation sites of bullous scabies.^[4,8]^

#### 2.1.5. Nail scabies.

Nail scabies has mostly been manifested in cases of crusted scabies in immunocompromised adults, but in rare cases, it has also been reported in healthy children and adults.^[3]^ Although extremely rare, isolated nail involvement without other dermatological manifestations of scabies has been reported.^[8]^ Fingernails and toenails are often discolored, thick, and dystrophic and may have subungual involvement.^[6,8]^

Nail scabies poses a question about how the scabies mites are reached under the nails.^[3]^ One hypothesis suggests that *S scabies*, which is found under nails as a result of scratching, gradually causes subungual hyperkeratosis, which would afterward lift the nail and weaken it, encouraging onycholysis and onychoschizia.^[3]^ Another hypothesis proposes that nail involvement results from chronic pruritus, which incites nail lesions by auto-inoculation.^[3]^

**Figure 1.**
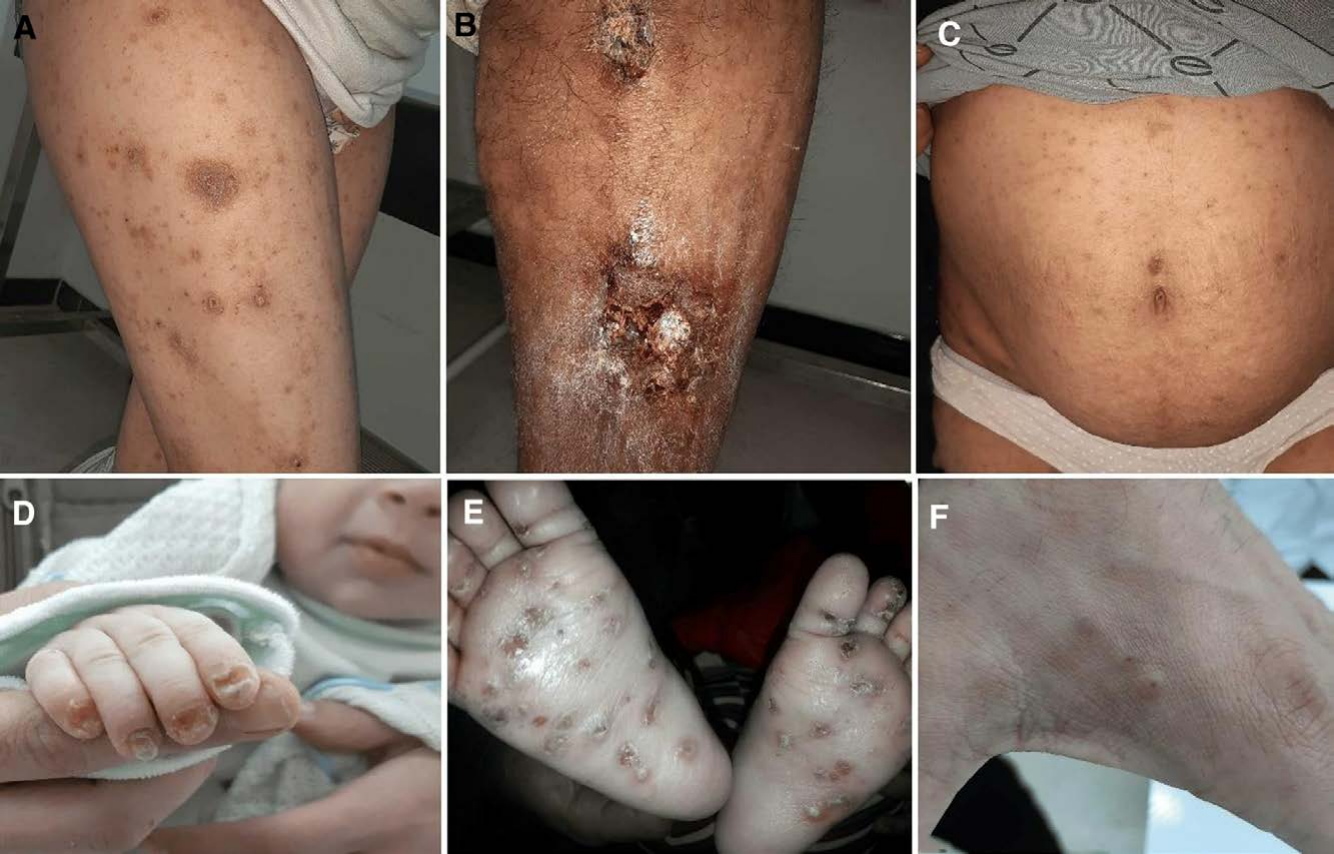
(A) Papulo-nodular and impetigo lesions on the leg of a 15-yr-old patient. (B) Scabies lesions affecting the leg with large erosions and scratches in a 40-yr-old male patient who applied an unknown topical treatment. (C) Numerous erythematous papules on the trunk of a 35-yr-old pregnant woman. (D) Nail scabies that presents as onycholysis of nail plates in a 2-mo-old infant. (E) Multiple crusted papules on the soles of the same 2-mo-old infant. (F) Nodules on the dorsal hand of scabies patient that persist after treatment.

Figure 1 shows some of the clinical manifestations and variants of scabies.

### 2.2. Scabies and affected age groups

#### 2.2.1. Scabies in infants and children.

Infants frequently present with severe rashes, irritability, inadequate nutrition, and failure to thrive.^[14]^ Scabies may be more recalcitrant in this age group, and the lesions typically occur more frequently than in older children and adults.^[14]^ In infants, scabies typically manifests as papules, vesicles, nodules, or pustules.^[14]^ On the other hand, burrows, erythematous papules, and inflammatory nodules are the most common manifestations of scabies in children, often accompanied by secondary excoriation.^[2]^ The palms, soles, face, neck, and scalp are more commonly involved locations in infants and children.^[7]^ The pruritus, in children younger than 1 year, may be absent or overlooked.^[3]^ Nodular or bullous scabies are less common in children with a possibility of developing vesicular, papulovesicular, or papulopustular eruptions in children younger than 2 years.^[2,8]^

#### 2.2.2. Scabies in elderly.

Atypical findings are common in the elderly, and although the inflammatory response may be reduced, pruritus often persists.^[6]^ Unlike adults, the scalp and face are common locations of scabies.^[4]^ Nevertheless, the scalp can also be affected in infants, children, and immunocompromised individuals.^[6]^

Old patients are more likely to develop crusted scabies; This is because of the presence of many factors related to this age group such as a different immune response, a lack of nutrition, a decline in cognitive function, and not being able to keep their personal hygiene.^[4]^

## 3. Diagnosis

### 3.1. History and examination

The diagnosis of scabies is usually established clinically and based on patient history.^[7,15]^ A history of pruritus in family, friends, or intimate contacts is helpful.^[13]^ Skin lesions can be seen on examination in a typical distribution, and characteristic serpiginous burrows can be seen with the naked eye.^[7]^

To assist physicians in the diagnosis of scabies, the 2020 International Alliance for the Control of Scabies consensus criteria as a standardized tool.^[2]^

These criteria present 3 categories of diagnosis: Confirmed scabies, clinical scabies, or suspected scabies.^[2]^ These criteria are summarized in (Table 2).^[2,16]^

**Table 2:** Summary of 2020 IACS criteria for the diagnosis of scabies.

A. Confirmed scabies
At least 1 of:
A1: Mites, eggs, or feces on light microscopy of skin samples
A2: Mites, eggs, or feces visualized on an individual using a high-powered imaging device
A3: Mites visualized on an individual using dermoscopy
B. Clinical scabies
At least 1 of:
B1: Scabies burrows
B2: Typical lesions affecting male genitalia
B3: Typical lesions in a typical distribution and 2 history features
C. Suspected scabies
One of:
C1: Typical lesions in a typical distribution and 1 history feature
C2: Atypical lesions or atypical distribution and 2 history features
H. History features
H1: Pruritus
H2: Close contact with an individual who has pruritus or typical lesions in a typical distribution

The diagnosis can be established at 1 of the 3 levels (A, B, or C). The diagnosis of suspected scabies or clinical scabies should only be obtained when other differential diagnoses are considered with less likelihood than scabies.

IACS = International Alliance for the Control of Scabies.

Some noninvasive diagnostic techniques such as dermatoscopy, videodermatoscopy, in vivo reflectance confocal microscopy (RCM), and optical coherence tomography (OCT) can help to confirm the diagnosis of scabies.^[5]^

### 3.2. Noninvasive imaging techniques

#### 3.2.1. Dermoscopy.

Dermoscopy, with ×10 magnification, has been considered a useful tool for diagnosing scabies by observing scabies burrows.^[5,17]^ The “delta wing jet” (also called “delta glider” or “jet with contrail”) sign on dermoscopy refers to scabies’ burrows which are seen as a white structureless line with a dark brown triangular structure, which corresponds to the head and anterior legs of the scabies mite, at the end of the burrow.^[4,7,17]^ In addition, eggs can be observed as small ovoid structures within the burrow.^[7]^

The “mini triangle” sign, which refers to the head of the maturing larva within the scabies egg, is another sign that has been seen in dermoscopy.^[4,8]^

The scabies mite is difficult to recognize through dermoscopy in darker skin phototypes, hairy areas, and complications incited by scratching (such as excoriations, bleeding, crusts, or small dirt particles).^[5,13]^ In crusted scabies, dermoscopy reveals several burrows and a hyperkeratotic appearance.^[4]^

#### 3.2.2. Videodermoscopy (VD).

VD is a noninvasive diagnostic tool that provides up to ×1000 magnification.^[4,6]^

When dermoscopy is unable to differentiate burrows from excoriations, the higher magnification of VD is considered helpful due to its high sensitivity and specificity.^[4,6]^

VD examines the skin surface down to the superficial dermis; Hence, it allows in vivo identification of burrows (at low magnifications ranging from ×40 to ×100) and scabies mites, larvae, eggs, or feces (at higher magnifications up to ×600).^[5]^

There are many advantages of using VD compared to other diagnostic tools such as skin scraping.^[5]^ It is a non-traumatic screening and does not cause any discomfort to the patients; Thus, it can be performed in children and non-cooperative patients.^[4,5]^ It can be performed quickly and easily, allowing examination of the entire skin surface typically within a few minutes.^[5]^ Also, it reduces cross-infection risk.^[4]^

VD is especially useful for post-therapeutic follow-up, in cases of unsuccessful treatment, because it exhibits the possibility of viable mites and reduces the likelihood of persisting and spreading the infestation.^[4,5]^

#### 3.2.3. Reflectance confocal microscopy.

RCM is a noninvasive diagnostic tool that uses a reflected laser beam to scan the various layers of the skin.^[4]^

RCM allows a thorough in vivo examination of adult mites, larval, larvae, eggs, and feces.^[4,5]^ It provides a glimpse into the actual mites’ behavior and demonstrates their real-time crawling within the burrow.^[5]^

The burrows are seen as a linear segment amidst the surrounding epidermis which is exhibited as a “honeycomb” pattern.^[4]^ The lack of availability, time-consuming (approximately 10 minutes for each lesion), and high cost of RCM limit the utilization of this tool.^[4,6]^

#### 3.2.4. Optical coherence tomography.

OCT is a noninvasive technique that is similar to ultrasonography, by using reflected near-infrared beams, but it has a higher resolution and shows the main components of the skin.^[4–6]^ Mites, eggs, feces, and burrows can all be identified using this technique.^[6]^

Due to the ability to identify the mite both vertically and horizontally, OCT can quickly and accurately diagnose scabies in vivo.^[5]^ In addition, OCT can be helpful for studying mite biology and monitoring the treatment progress.^[5]^

#### 3.2.5. Burrow ink test (BIT).

The BIT allows for the visualization of a typical curvilinear burrow structure by applying ink to visible burrows on the skin and then removing the excess ink with alcohol.^[2,4]^

BIT is considered positive after seeing a dark and zigzagged line with the naked eye after wiping the ink off with alcohol.^[4]^ Anyhow, this procedure provides a partial diagnosis and it cannot distinguish old from new lesions.^[1]^

#### 3.2.6. Adhesive tape test.

Adhesive tape test includes the application of adhesive tape to suspicious skin lesions.^[2]^

After applying the adhesive tape to the skin, it is pulled off and immediately transferred onto a slide to look for evidence of scabies on microscopic examination.^[2,4]^

### 3.3. Invasive techniques

#### 3.3.1. Skin scrapings with microscopy.

The diagnosis of scabies is confirmed through microscopic examination of mites, eggs, or feces from obtained scales by skin scraping.^[2,11]^

A drop of mineral oil is applied to the suspected lesions, then a sterile blade is used to scrape the lesion gently.^[2,11]^ However, this procedure is time-consuming, requires laboratory facilities, and may cause accidental infections.^[5,7]^ Also, skin scraping may not be well tolerated, especially by young patients.^[5,7]^

#### 3.3.2. Skin biopsy.

Scabies diagnosis based on histopathology is considered to be one of the most accurate tests.^[1]^ If a skin biopsy is taken at the location of a burrow will reveal the mite and its products.^[13]^

The histopathology is not considered a part of routine work-up for the diagnosis of scabies and is saved for the confirmation of atypical manifestations.^[1]^ However, it is a time-consuming and expensive procedure and may require 2 to 7 days to obtain a result using routine fixation processes.^[1,13]^

#### 3.3.3. Serology.

There are no available standardized laboratory tests for diagnosing scabies.^[7]^ Although several candidate antigen and antibody immunoassays have been evaluated, none have been widely adopted due to subpar results.^[7]^

The enzyme-linked immunosorbent assay (ELISA) detects the antibodies of *S scabiei*, which are produced prior to the onset of clinical symptoms.^[5]^ In addition, high IgE levels were reported in scabies patients, by using inhibition ELISA.^[1]^ ELISA is an extremely sensitive and accurate diagnostic tool but its disadvantages include being time-consuming, not currently available, and requiring a microplate reader.^[5,18]^

Tyrosine kinase of *S scabiei*, which is mostly found in the oral cavity of scabies mites, is a cloned protein that has been investigated also for scabies diagnosis with an ability to detect infection early in its course^.[1]^

#### 3.3.4. Modern molecular techniques.

The DNA of *S scabiei* can be extracted using various procedures including swab specimens and skin biopsies^.[1]^

##### 3.3.4.1. Polymerase chain reaction (PCR) assay.

Nested-PCR based on the C-oxidase subunit (cox1) gene of *S scabiei* is considered a highly sensitive test for scabies diagnosis.^[4,7]^

Although PCR is considered a specific and sensitive tool, it requires specialized equipment.^[18]^ Also, it is unable to detect genes in cases where the mite DNA is absent from the infected skin area.^[18]^

However, PCR assays are considered impractical, because the testing was not performed in all forms of scabies such as in elderly patients and crusted Scabies.^[1]^ The possibility of using this is as a screening during scabies outbreaks.^[1]^

##### 3.3.4.2. Isothermal amplification techniques.

Despite the use of PCR-based identification tools, thermocyclers must still be used to amplify the DNA.^[1]^ In order to address this issue, isothermal amplification techniques have been developed.^[1]^

These techniques include rolling circle amplification (RCA), multiple displacement amplification (MDA), and loop-mediated isothermal amplification (LAMP).^[1]^ It is possible to identify a wide variety of microorganisms using these techniques.^[1]^

## 4. Treatment and prevention

To avoid transmission and reinfestation, all close contacts must be treated simultaneously, even if they are asymptomatic. They may be a source of reinfestation for others if they are not treated.^[8,13]^

To minimize the disease transmission, bed sheets and clothes used during the 3 days before treatment should be machine-washed with hot water (≥50 C or 122 °F) and dried in a hot dryer, but sterilization is not needed.^[8,13]^

Alternatively, clothing and other similar items or difficult-to-exterminate items can be preserved for at least 72 hours in a sealed plastic bag.^[2,13]^ This procedure is especially significant in cases of crusted scabies.^[8]^ However, there is no vaccine that can prevent scabies.^[13]^

If there are no signs of active scabies 1 week after treatment is over, the infestation is considered cleared.^[19]^

The diagnosis of treatment failure should not be made until at least 6 weeks after treatment has been completed, because it can take this long for signs and symptoms of hypersensitivity to resolve.^[7]^ Post-treatment itching may persist for 1 to 4 weeks, even after effective treatment.^[6,19]^

After treatment, persistent signs and symptoms may occur due to many causes such as incorrect diagnosis, reinfection, prescription of an inappropriate drug, noncompliance or improper application of the topical treatment or its local reaction, post-scabietic reaction to the mite or its products, delusions of parasitosis, and resistance.^[20]^

The use of emollients, oral antihistamines, and low-potency topical corticosteroids can be efficient in post-scabietic pruritus.^[6]^ Although histamine is not considered to be the primary mediator implicated in scabies pruritus, antihistamines, primarily sedatives, may help patients to sleep.^[15]^

The older first-generation H1-antihistamines can easily cause sedation, fatigue, drowsiness, and impairment of concentration and memory.^[21]^ Therefore, their use should be discouraged.^[21]^ On the other hand, the newer second-generation H1-antihistamines are safer, cause less sedation, and are more effective.^[21]^

High-potency or oral corticosteroids are not recommended due to the probable side effects.^[6]^

### 4.1. Management of classic scabies

The type of scabies, patient age, reported efficacy of various treatments, and side effect profile all determine the choice of treatment.^[4]^ A potent acaricide agent should be administered orally or topically to the patients.^[22]^

Permethrin 5% cream is the first-line topical medication in the United Kingdom and the United States.^[7]^ On the other hand, the world health organization listed ivermectin as of the safest and most efficient therapeutic drugs required in a healthcare system.^[13]^

#### 4.1.1. Permethrin.

Permethrin is the first-line topical treatment in western countries and is preferred over other topical treatments.^[7,22]^ It is highly effective after a single application due to the adulticidal and ovicidal against the scabies mite.^[7]^ However, in practice, 2 applications are frequently included in the prescribed regimen.^[7]^

Permethrin hyperpolarizes parasitic cell membranes by binding to and stabilizing voltage-gated sodium channels.^[23]^

Permethrin 5% cream is applied overnight from the scalp to toes, avoiding the skin of periorbital and perioral locations, and washed off after 8 to 12 hours and then it must be repeated after 7 to 14 days.^[14,19]^

The second application is used to eliminate newly hatched mites, as the first application is not always effective in eliminating all the eggs.^[13]^

Permethrin 5% cream is FDA-approved and is safe during pregnancy and lactation and in children at the age of 2 months and older.^[8,24]^ However, this drug may provoke neurological complications.^[25]^ Thus, a briefer duration of application is encouraged in infants <2 months of age.^[25]^

Adverse effects are reported infrequently, generally mild, and confined to local cutaneous reactions like transient paresthesia, irritation, erythema, dryness, burning sensation, pruritus, eczema, and contact dermatitis.^[7,8,14]^

#### 4.1.2. Ivermectin.

Scabies can be treated with ivermectin orally.^[7]^ Due to a possible lack of ovicidal effect, the recommended dosage is 1 oral dose (taken with food) of 200 mcg/kg of body weight and repeated on day 14.^[19,22]^

The standard treatment, 2 doses 2 weeks apart, has a cure rate close to 100%, similar to that of topical 5% permethrin.^[7]^

In muscle and nerve cells, ivermectin binds specifically to glutamatergic chloride channels, causing the scabies mite to become paralyzed and die from hyperpolarization and increased cell membrane permeability.^[2]^

Oral ivermectin has a quick and simple administration and is more effective than lindane and crotamiton.^[23]^

Ivermectin has been shown to be effective as a mass treatment in controlling both endemic and epidemic scabies.^[26]^ It is usually cheaper and easier to administer in large outbreaks than topical therapies.^[13]^

Ivermectin is approved in several countries, but it has not been approved by the FDA.^[6]^

Ivermectin is not recommended for use in pregnant women or young children (<5 years old or 15 kg) due to insufficient safety data.^[6]^

Infants with severe secondary eczema, erosions, or ulcers who have scabies may show an effective treatment with oral ivermectin, as topical therapies are likely to have serious cutaneous and systemic side effects.^[14]^

Several studies have demonstrated that topical ivermectin has comparable cure rates and is equally efficient against scabies in comparison to topical permethrin.^[8]^

In classic scabies, preliminary studies demonstrate that topical ivermectin 1% lotion is safe and effective as oral ivermectin but it is not used frequently due to its cost, availability, and lack of large-scale evidence of its usage.^[2]^

The usage of oral ivermectin in infants and children who weigh <15 kg is not approved, due to the limited safety data on its administration in children who weigh <15 kg.^[27]^

Side effects of oral ivermectin include headache, nausea, vomiting, dizziness, asthaenia, paraesthesia, hypotension, fever, chills, anorexia, rash, pruritus, edema, dyspnea, abdominal pain, gastrointestinal upset, myalgia, and arthralgia.^[8,13,14]^ However, the majority of ivermectin-related side effects are mild and transient.^[9]^

#### 4.1.3. Sulfur.

Sulfur is a topical treatment for scabies.^[7]^ Sulfur compounds, containing 5% to 10% sulfur, are effective when used for successive 3 days.^[7,13]^ Also, the preparations of 5% to 10% sulfur in paraffin are widely used in Africa and South America and are considered safe for pregnant women and infants.^[7,13]^

Sulfur transforms into hydrogen sulfide, polythionic acid, and pentathion on the skin, which has antibacterial properties and has been shown efficient to exterminate scabies mites.^[2]^

In infants younger than 2 months, Preparations containing 5% sulfur are recommended as an alternative therapeutic option to permethrin.^[2,14]^ Sulfur ointment is messy has an unpleasant odor and can cause skin irritation, but it is effective and safe.^[13]^ Anyhow, sulfur is not approved by the FDA.^[13]^

#### 4.1.4. Benzyl benzoate.

Benzyl benzoate, an ester of benzyl alcohol and benzoic acid, is a very effective anti-scabietic treatment with excellent cure rates and has been used in many countries, including in Europe and Australia.^[7]^

Benzyl benzoate lotion 10% to 25% is applied once daily at night for 2 successive days and must be repeated after 7 days.^[19]^

Benzyl benzoate should be diluted to 6.25% for infants and 12.5% for children due to its adverse effects such as skin irritation and burning sensation.^[2,19]^ Therefore, it should not be used in children under the age of 2.^[25]^

Benzyl benzoate is classified as Category C in pregnancy and has been banned in the United States.^[28]^

#### 4.1.5. Lindane.

Lindane is an efficient scabicidal organic insecticide with robust anti-scabietic effects.^[7]^

Due to its significant systemic absorption and subsequent toxicity to the central nervous system, Lindane is no longer recommended.^[2,19]^

Nausea, vomiting, restlessness, disorientation, tremor, seizures, and even death have all been reported as neurotoxic side effects following topical application.^[7]^ As a result, the drug is no longer being sold in many countries.^[7]^ However, lindane must be avoided in infants, immunocompromised patients, and pregnant and breastfeeding women.^[2,7]^

#### 4.1.6. Crotamiton.

Crotamiton cream 10% has an unknown mechanism of action and has been preferred in infants and children because of its low toxicity; However, to achieve a satisfactory response, it typically requires multiple applications due to its limited efficacy.^[2,7]^ Crotamiton is approved by FDA for adults but not approved for children.^[24]^

It should be applied to the entire body for 24 hours, washed off, and then reapplied for 3 to 5 days.^[2]^ Some studies suggest that daily application for 10 to 14 days may increase treatment efficacy.^[2]^

Side effects are uncommon and may include methemoglobinemia in children and allergic contact dermatitis.^[2]^

#### 4.1.7. Malathion.

Malathion lotion 0.5% prevents acetylcholine from being degraded by inhibiting the activity of cholinesterase^.[2]^ However, there is limited evidence that demonstrates that it is effective in scabies treatment.^[2]^

The safety of pregnant women who were treated with malathion was not studied.^[19]^

#### 4.1.8. Moxidectin.

Oral moxidectin may be sufficient to eliminate the infestation with a single dose due to its longer half-life (more than 20 days compared to 14 hours for ivermectin).^[7]^ It has better skin retention and appears to prevent reinfestation for a longer period after treatment compared to ivermectin. Thus, it does not require a second dose.^[4,7]^

#### 4.1.9. Fluazuron.

Fluazuron is a benzoylphenyl urea compound that blocks the synthesis of chitin and inhibits the arthropod nervous system involving the scabies mite.^[7,23]^

It has no efficacy against adult mites, but it prevents new larvae from growing within the eggs.^[7]^

The isoxazolines, which include afoxolaner and fluralaner, another promising oral drug class, demonstrated efficiency in animal models as single-dose regimens.^[6]^ Anyhow, the FDA issued an alert regarding the potential neurotoxicity of these agents.^[6]^

Other botanical products utilized with varying results of treatment include tea tree oil, turmeric, Lippia and neem oils, and clove.^[7]^

### 4.2. Management of crusted scabies

Crusted scabies treatment requires a combination of oral and topical therapeutic options to reduce the high mite burden and penetrate thick scales.^[6]^

The preferred first-line treatment for crusted scabies is a combination of oral ivermectin (200 mcg/kg as a single dose, given on days 1, 2, 8, 9, and 15) and topical 5% permethrin cream or benzyl benzoate (daily for 7 days, then weekly until cure).^[8]^

Oral ivermectin in 3, 5, or 7 standard doses, topical permethrin or benzyl benzoate every 2 to 3 days for 1 to 2 weeks, and a topical keratolytic (such as the use of 10% urea and 5% lactic acid) are all suggested by the Centers for Disease Control and Prevention (CDC) for crusted scabies treatment.^[6,8]^

Ivermectin is taken orally at a dose of 200 mcg/kg, and the duration of treatment is determined by the severity. The following are the recommended treatments of ivermectin based on the severity of crusted scabies: Grade 1 (3 doses over 1 week), grade 2 (5 doses over 2 weeks), and grade 3 (7 doses over 4 weeks).^[4,13]^

The resolution of the lesion may be accelerated by the mechanical removal of the hyperkeratotic layers.^[8]^

### 4.3. Management of nodular scabies

Post-scabetic localized nodules after eradication of scabies mites can be treated with a high potency topical corticosteroid, intralesional injection of a corticosteroid (such as triamcinolone), short-course oral prednisolone, tacrolimus, pimecrolimus, or liquid nitrogen cryotherapy.^[4,8]^

### 4.4. Management of bullous scabies

Systemic and topical treatments for bullous scabies are comparable to those for classical scabies.^[26]^ Oral ivermectin is an option for patients who have a poor response or an allergic reaction to topical treatment.^[26]^ Oral prednisolone may aggravate the lesions in rare conditions.^[26]^ Multiple applications must be administered for recurrent bullous scabies.^[26]^

### 4.5. Management of nail scabies

Nail scabies is difficult to be treat and is not highly based on evidence.^[3]^

Nail trimming, debridement of subungual material, and rehashed utilization of scabicide are recommended.^[8,22]^

### 4.6. Management of secondary bacterial skin infections

Topical antibiotics, like mupirocin, fusidic acid, and retapamulin may be required for the treatment of secondary bacterial skin infection.^[8]^

The treatment of severe secondary bacterial infections may require systemic treatment with clindamycin, cloxacillin, macrolides, or first- or second-generation cephalosporins.^[8]^

Children with secondary bacterial infection (moderate, severe, or refractory to topical agents) can be treated with empirical antibiotic therapy, guided by local patterns of sensitivity with an emphasis on the methicillin-resistant staphylococcus aureus prevalence.^[2]^ in communities where this is endemic, an empiric cover for methicillin-resistant staphylococcus aureus is required.^[2]^

## 5. Conclusion

Scabies is a common contagious parasitic infestation that is considered a global public health issue in all nations regardless of social and economic status.

Dermatologists should be aware of all the available diagnostic and therapeutic techniques. Increased awareness of various presentations/variants of scabies may lead to a more accurate and faster diagnosis. The early diagnosis and treatment of scabies are important to prevent complications and reduce transmission.

## Author contributions

**Supervision:** Jacob Al-Dabbagh.

**Validation:** Razan Younis, Nemat Ismail.

**Writing – original draft:** Jacob Al-Dabbagh.

**Writing – review & editing:** Jacob Al-Dabbagh, Razan Younis, Nemat Ismail.

## References

[1] Siddig EE Hay R. Laboratory-based diagnosis of scabies: a review of the current status. Trans R Soc Trop Med Hyg. 2022;116:4–9. 33763705 10.1093/trstmh/trab049 PMC8776561

[2] Thompson R Westbury S Slape D. Paediatrics: how to manage scabies. Drugs Context. 2021;10:2020-12-3. 10.7573/dic.2020-12-3 PMC8007207 33828606

[3] Chinazzo M Desoubeaux G Leducq S. Prevalence of nail scabies: a French prospective multicenter study. J Pediatr. 2018;197:154–7. 29576324 10.1016/j.jpeds.2018.01.038

[4] Arora P Rudnicka L Sar-Pomian M. Scabies: a comprehensive review and current perspectives. Dermatol Ther. 2020;33:e13746. 32484302 10.1111/dth.13746

[5] Micali G Lacarrubba F Verzì AE. Scabies: advances in noninvasive diagnosis. PLoS NeglTrop Dis. 2016;10:e0004691. 10.1371/journal.pntd.0004691 PMC4911127 27311065

[6] Thomas C Coates SJ Engelman D. Ectoparasites: scabies. J Am Acad Dermatol. 2020;82:533–48. 31310840 10.1016/j.jaad.2019.05.109

[7] Chandler DJ Fuller LC. A review of scabies: an infestation more than skin deep. Dermatology. 2019;235:79–90. 30544123 10.1159/000495290

[8] Leung AKC Lam JM Leong KF. Scabies: a neglected global disease. Curr Pediatr Rev. 2020;16:33–42. 31544694 10.2174/1573396315666190717114131

[9] Motswaledi HM. Clinical diagnosis and treatment of scabies, a neglected tropical disease. S Afr Fam Pract (2004). 2021;63:e1–6. 10.4102/safp.v63i1.5224 PMC8378203 34342482

[10] Fischer K Holt D Currie B. Scabies: important clinical consequences explained by new molecular studies. Adv Parasitol. 2012;79:339–73. 22726646 10.1016/B978-0-12-398457-9.00005-6

[11] Sánchez-Borges M González-Aveledo L Capriles-Hulett A. Scabies, crusted (Norwegian) scabies and the diagnosis of mite sensitisation. Allergol Immunopathol (Madr). 2018;46:276–80. 29279260 10.1016/j.aller.2017.05.006

[12] Kim HS Hashimoto T Fischer K. Scabies itch: an update on neuroimmune interactions and novel targets. J Eur Acad Dermatol Venereol. 2021;35:1765–76. 33960033 10.1111/jdv.17334

[13] Richards RN. Scabies: diagnostic and therapeutic update. J Cutan Med Surg. 2021;25:95–101. 32998532 10.1177/1203475420960446

[14] Lobo Y Wheller L. A narrative review of the roles of topical permethrin and oral ivermectin in the management of infantile scabies. Australas J Dermatol. 2021;62:267–77. 34184244 10.1111/ajd.13654

[15] Jannic A Bernigaud C Brenaut E. Scabies itch. Dermatol Clin. 2018;36:301–8. 29929601 10.1016/j.det.2018.02.009

[16] Engelman D Yoshizumi J Hay RJ. The 2020 international alliance for the control of scabies consensus criteria for the diagnosis of scabies. Br J Dermatol. 2020;183:808–20. 32034956 10.1111/bjd.18943 PMC7687112

[17] Ueda T Katsura Y Sasaki A. Gray-edged line sign of scabies burrow. J Dermatol. 2021;48:190–8. 33063894 10.1111/1346-8138.15650 PMC7894142

[18] Akuta T Minegishi D Kido N. Development of a rapid scabies immunodiagnostic assay based on transcriptomic analysis of *Sarcoptes scabiei* var. nyctereutis. Sci Rep. 2021;11:6455. 33742008 10.1038/s41598-021-85290-7 PMC7979781

[19] Salavastru CM Chosidow O Boffa MJ. European guideline for the management of scabies. J Eur Acad Dermatol Venereol. 2017;31:1248–53. 28639722 10.1111/jdv.14351

[20] Khalil S Abbas O Kibbi AG. Scabies in the age of increasing drug resistance. PLoS NeglTrop Dis. 2017;11:e0005920. 10.1371/journal.pntd.0005920 PMC5708620 29190303

[21] Church MK Church DS. Pharmacology of antihistamines. Indian J Dermatol. 2013;58:219–24. 23723474 10.4103/0019-5154.110832 PMC3667286

[22] Bouvresse S Chosidow O. Scabies in healthcare settings. Curr Opin Infect Dis. 2010;23:111–8. 20075729 10.1097/QCO.0b013e328336821b

[23] Inam W Walton S Khan S. Molecular drug targets for scabies: a medicinal chemistry perspective. Future Med Chem. 2020;12:2225–38. 33243012 10.4155/fmc-2020-0182

[24] Hill TA Cohen B. Scabies in babies. Pediatr Dermatol. 2017;34:690–4. 28833468 10.1111/pde.13255

[25] Kazeminejad A Hajheydari Z Ghahari MJ. Scabies treatment in children: a narrative review. J Pediatr Rev. 2019;7:105–12.

[26] Luo DQ Huang MX Liu JH. Bullous scabies. Am J Trop Med Hyg. 2016;95:689–93. 27402514 10.4269/ajtmh.16-0273 PMC5014280

[27] Levy M Martin L Bursztejn AC. Ivermectin safety in infants and children under 15 kg treated for scabies: a multicentric observational study. Br J Dermatol. 2020;182:1003–6. 31344258 10.1111/bjd.18369

[28] Patel VM Lambert WC Schwartz RA. Safety of topical medications for scabies and lice in pregnancy. Indian J Dermatol. 2016;61:583–7. 27904173 10.4103/0019-5154.193659 PMC5122270

